# Tumor suppressor Nf2/merlin drives Schwann cell changes following electromagnetic field exposure through Hippo-dependent mechanisms

**DOI:** 10.1038/cddiscovery.2015.21

**Published:** 2015-09-07

**Authors:** A Colciago, S Melfi, G Giannotti, V Bonalume, M Ballabio, L Caffino, F Fumagalli, V Magnaghi

**Affiliations:** 1 Dipartimento di Scienze Farmacologiche e Biomolecolari, Università degli Studi di Milano, Via G. Balzaretti 9, Milan 20133, Italy

## Abstract

Previous evidence showed mutations of the neurofibromin type 2 gene (*Nf2*), encoding the tumor suppressor protein merlin, in sporadic and vestibular schwannomas affecting Schwann cells (SCs). Accordingly, efforts have been addressed to identify possible factors, even environmental, that may regulate neurofibromas growth. In this context, we investigated the exposure of SC to an electromagnetic field (EMF), which is an environmental issue modulating biological processes. Here, we show that SC exposed to 50 Hz EMFs changes their morphology, proliferation, migration and myelinating capability. In these cells, merlin is downregulated, leading to activation of two intracellular signaling pathways, ERK/AKT and Hippo. Interestingly, SC changes their phenotype toward a proliferative/migrating state, which in principle may be pathologically relevant for schwannoma development.

## Introduction

Neurofibromin 2 gene (*Nf2*) encodes the tumor suppressor protein merlin, a cytoskeleton-associated protein belonging to the ERM (ezrin-radixin-moesin) family. Mutations in the *Nf2* gene origin an autosomal dominant multiple syndrome called neurofibromatosis type 2,^1^ leading to merlin loss and determining the transformation of Schwann cells (SCs) into a form of benign tumor called schwannoma. About 5% of vestibular schwannomas (which are neurofibromas originating from the SC of the eighth cranial vestibular nerve) are related to inherited *Nf2* mutations, whereas the remaining 95% are sporadic.^2^ Indeed, between 30 and 50% of new cases arise by random genetic mutations.^3^ However, merlin is mutated at least in 66% of sporadic schwannomas indicating a correlation between this protein and tumor development.^4^

Recent research on *Nf2* gene showed that merlin is involved in different signal transduction pathways, including MAPK/ERK and Hippo pathways.^5,6^ Indeed, in merlin null schwannomas the MAPK/ERK pathway is activated,^6^ whereas the Hippo pathway is involved in several cell transformations,^7^ mostly through control of the transcriptional co-activator Yes-associated protein (Yap).^8^ Of note, Hippo has been indicated as a major effector of merlin in growth regulation,^9^ even though this correlation was poorly investigated in SCs.

In the last years, some efforts have been addressed to determine possible factors, even environmental, that may regulate neurofibromas growth. In this context, the exposure to electromagnetic fields (EMFs) is an environmental issue modulating biological processes in different type of cells. EMF emissions yield changes in migration, cytoskeleton organization, ion channels regulation and oxidative balance.^10–13^ Some epidemiologic studies indicate a consistent risk for the occurrence of neurodegenerative diseases and different tumors,^14^ including schwannomas, in subjects exposed to EMFs. Data from clinical case–control studies corroborate this pathogenic correlation.^15–18^ Nevertheless, EMF is used in clinic to treat different pathologic conditions, indeed the therapeutic use of low-frequency (20 Hz) EMFs has long been proposed as a reliable tool to promote re-myelination and peripheral nerve regeneration.^19–21^ However, variable biologic responses to the different frequency exposures have been hypothesized. Mechanisms correlating the EMF exposure to the biological changes in SC, likely to the onset of schwannomas and the possible alterations in merlin, were far to be elucidated.

The aim of the present study was to investigate whether the tumor suppressor merlin may regulate cellular and biochemical features, such as morphology, viability, proliferation, and myelinating capability of SC exposed to EMFs. Our results showed that EMF at higher frequency (50 Hz) regulates SCs phenotype and likely affects their pathophysiologic condition, by activation of ERK/AKT and Hippo signaling pathways.

## Results

### Exposure to EMF induces changes in SCs morphology and proliferation

**Figure 1:**
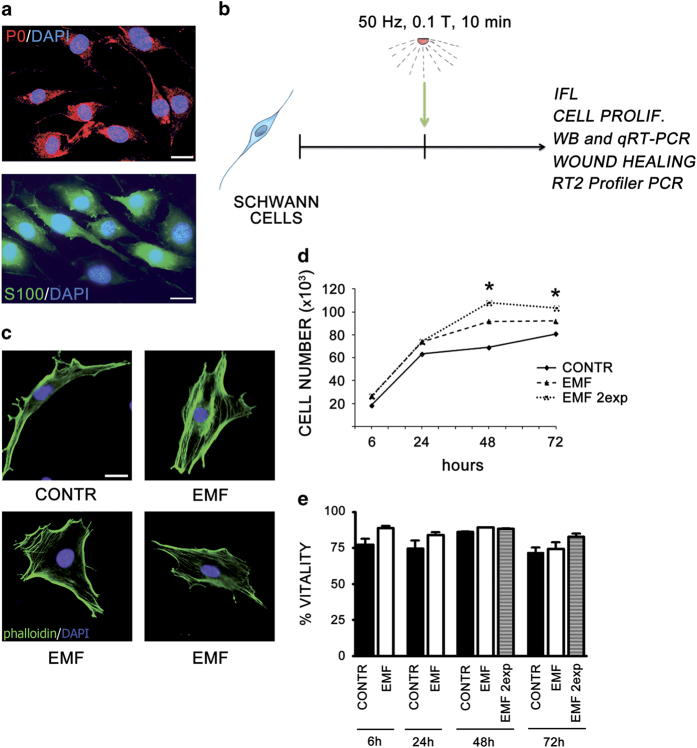
SCs change morphology and proliferation following EMF exposure. (**a**) Merge image of SC characterized by immunopositivity for P0 and S100 markers, respectively (anti-P0-594, in red; anti-s100-488, in green), showing a cell purity more than 98%. Nuclei were stained with DAPI, in blue. Scale bar 10 *μ*m. (**b**) Scheme of the experimental model used. SCs were exposed to EMF of 50 Hz, 0.1 T, for 10 min, then cells were assayed for proliferation, migration, vitality, chemoresponsivity, morphology, western blot, qRT-PCR and RT^2^ profiler PCR. (**c**) EMF exposure induces SCs morphologic rearrangements in actin cytoskeleton, as assessed by immunopositivity for f-actin (phalloidin-FICT, in green). SCs turned from a spindle-shaped to an enlarged phenotype, suggesting alterations in the differentiation program. Nuclei were stained with DAPI, in blue. Scale bar 10 *μ*m. (**d**) SCs proliferation was assessed at 6, 24, 48 and 72 h, following a single (dashed line) or double (dot line) EMF exposure. EMFs produced a significant (*P<0.05) increase in cell proliferation. Experiments were repeated at least three times and data expressed as cell number (×10^3^). Two-way ANOVA using Bonferroni’s *post hoc* test was used for statistical analysis. (**e**) Percentage of SCs vitality was assessed at 6, 24, 48 and 72 h, following a single (white columns) or double (gray columns) EMF exposure, but no significant changes in SCs vitality were observed. Controls (CONTR, black columns). The values are means±S.D. (*N*=3).

SCs primary cultures from 3-day-old rats were used for the experiments. These cells showed the characteristic spindle-shape morphology *in vitro*. Cell purity, more than 98%, was assessed performing immunolabeling for the typical markers myelin protein zero (P0) and protein S100 (Figure 1a). To test the effects of EMF on SC biological features, we applied an EMF intensity of 50 Hz, 0.1T for 10 min (Figure 1b). EMF exposure induced morphologic rearrangements in actin cytoskeleton, which might be critical for SCs differentiation and myelination. SCs turn from a spindle-shaped to an enlarged phenotype, indicating a dysregulation in the differentiation program; in fact, cells exposed to the treatment resemble to the undifferentiated phenotype (Figure 1c).

We next evaluated the effects of EMFs on cell proliferation to test whether EMF exposure may yield other SCs biological changes. Specifically, SCs count was evaluated *in vitro* at 6, 24, 48 and 72 h following EMF exposure (Figure 1d). The effect already present after 24 h became significantly evident at longer times, 48 and 72 h (*P*<0.05). Noteworthy, a second EMF exposure after 24 h strongly increases SCs proliferation at 48 and 72 h (Figure 1d), likely suggesting an additive effect of EMF on SCs proliferation. This effect was not dependent on a decrease in cell death. Indeed, EMF did not induce any sign of cell death or changes in cells viability at all time points considered, even after a double exposure (Figure 1e). However, the effect observed appeared cell specific, since proliferation of SaOS-2 cell (osteosarcoma) was not affected by EMF exposure (Supplementary Figure S1). Taken together, these findings suggested a SCs shift toward an undifferentiated state, mainly attributable to an altered control of cell proliferation.

### SCs exposed to EMFs possess greater migratory and chemotactic capabilities

**Figure 2:**
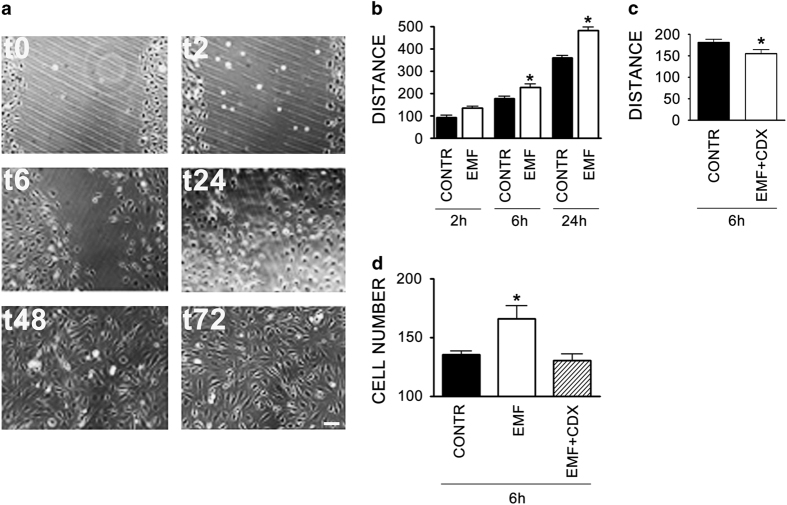
SCs migration and chemoresponsivity are increased following 10 min EMF exposure. (**a**) Microscopic images of SCs cultures, exposed to EMF, in which a scratch has been done on the bottom of the well (t0). Medium was replaced and cell proliferation was blocked by adding 50 ng/ml mitomycin. SCs migrate after 2 (t2) and 6 (t6) h, closing the wound region within 24 h (t24). A complete closure was seen at 48 (t48) and 72 (t72) h. Scale bar 10 *μ*m. (**b**) Histograms of cell distance (*μ*m) of SCs exposed to 10 min EMF (EMF, white columns) *versus* controls (CONTR, black columns). EMFs produced a significant (*P<0.05) increase in cell migration at 6 and 24 h. The distance (*μ*m) was calculated as difference between measurements of empty space at time 0 and following time points (2, 6 and 24 h). The values are means±S.D. (*N*=3). (**c**) Distance (*μ*m) of SCs exposed for 6 h to 10 min EMF was significantly counterbalanced (*P<0.05) by pre-treatment with cyclodextrin 5 mM (EMF+CDX, white column). Experiments and data were calculated *versus* controls (CONTR, black columns), as above. The values are means±S.D. (*N*=3). (**d**) A 10-min EMF exposure make the SCs significantly (*P<0.05) responsive to the chemotactic agent FCS 1%. Migrating cell number (per well) of SCs exposed (EMF, white column) was calculated after 6 h, *versus* controls (CONTR, black columns). SCs co-treated with 5 mM cyclodextrin reversed the chemotactic response to the control levels (diagonal lines column). The values are means±S.D. (*N*=3). One-way ANOVA using Tukey's post-test was used for all statistical analysis.

We used a wound-healing assay, performed on a cell monolayer, and Boyden chamber assay to measure motility. We found that EMF exposure promoted the SC motility. Indeed, a great number of SCs were able to repopulate the wounded region within 24 h, achieving a complete closure of the two sides at later times, 48 and 72 h (Figure 2a). EMF exposure induced a significant increase (*P*<0.05) in SC motility at 6 and 24 h, corroborating the EMF effect on cell migration. The concomitant presence of mitomycin 50 ng/ml excluded any proliferative component on the gap closure observed (Figure 2b). The effect was significantly counteracted (*P*<0.05) by a pre-treatment with cyclodextrin 5 mM (Figure 2c), a molecule used to produce changes in membrane cholesterol,^22^ thus indicating that a disorganization in lipid rafts and cell membrane architecture may alter the SC migratory response to the EMF. This is in accordance with the morphologic rearrangement in actin cytoskeleton seen in Figure 1c.

We next measured the SCs chemotactic response by mean of Boyden chamber assay. We found that EMF-exposed SCs were more responsive to a chemotactic agent, such as the fetal calf serum (FCS; Figure 2d). The co-treatment with 5 mM cyclodextrin abolished the chemotactic migration to the control levels. This effect strongly suggested the hypothesis that an autocrine factor, tightly interacting with the cell membrane, may regulate the SCs development, then addressing their fate toward a proliferative state and in turn changing their migration and chemotactic responsiveness.

### Expression of myelin proteins P0 and peripheral myelin protein of 22 kDa (PMP22) is changed in EMF-exposed SCs

**Figure 3:**
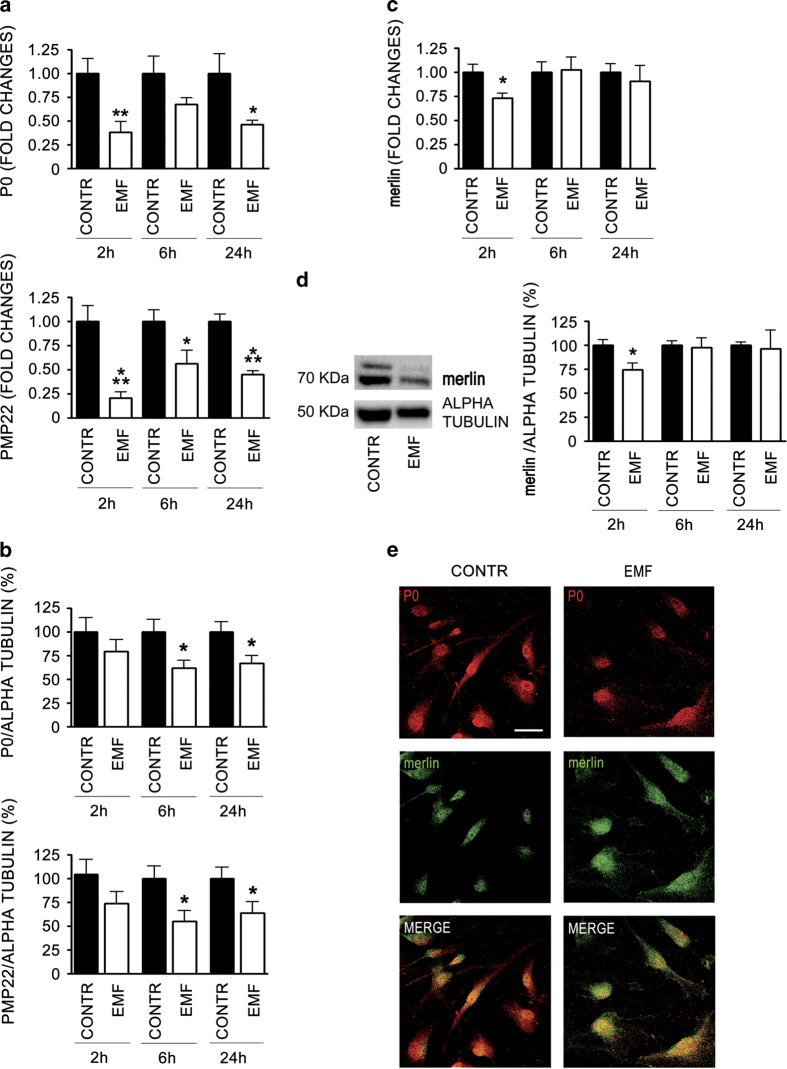
Myelin proteins (P0 and PMP22) and Nf2/merlin are decreased following 10 min EMF exposure. (**a**) Relative quantification by qRT-PCR of mRNA levels, coding for proteins P0 and PMP22 respectively, showed a decreased expression at all time points considered, 2, 6 and 24 h. Data were normalized to the housekeeping genes *α*-tubulin and *β*2-microglobulin and expressed as difference (^ΔΔ^Ct) *versus* controls, then averaged for each experimental group. The columns control (CONTR, black) and EMF-exposed SC (EMF, white) were expressed as fold changes. The values are means±S.D. (*N*=3). *P<0.05, **P<0.01, ***P<0.001. (**b**) Western blot analysis corroborated the decrease of P0 and PMP22 in EMF-exposed SCs (EMF, white columns), with significant effects at later times, 6 and 24 h (*P<0.05). Data were normalized for *α*-tubulin, were expressed as percentage *versus* controls (CONTR, black columns). The values are means±S.D. (*N*=3). (**c**) Neurofibromin 2 (Nf2) mRNA levels were assayed by qRT-PCR, showing a significant decrease (*P<0.05) 2 h after EMF exposure. Data normalized to *α*-tubulin and *β*2-microglobulin were expressed as difference (^ΔΔ^Ct) *versus* controls, then averaged for each experimental group. The columns control (CONTR, black) and EMF-exposed SC (EMF, white) were expressed as fold changes. The values are means±S.D. (*N*=3). (**d**) Accordingly, also the Nf2 protein levels were decreased 2 h following EMF exposure. Qualitative immunoblot (left panel) showing that the specific band for Nf2 (70 KDa) was downregulated in SCs. The *α*-tubulin (50 KDa) was used as a housekeeping protein. Quantitative data (right panel) confirmed a significant downregulation of Nf2/merlin at 2 h (*P<0.05, white columns), while no changes were observed at 6 and 24 h. Experiments were normalized for *α* -tubulin, and expressed as percentage *versus* controls (CONTR, black columns). The values are means±S.D. (*N*=3). One-way ANOVA using Tukey's post-test was used for all statistical analysis. (**e**) Confocal images showed a different cellular distribution of Nf2/merlin. SCs were immunopositive for the specific markers P0 (red) and Nf2 (green). Merge images (yellow) revealed that merlin was more localized in the cytoplasm of EMF-exposed SCs, rather than in the nucleus (CONTR). Scale bar 10 *μ*m.

As expected, the increase in proliferation/migration observed was associated with a decrease in myelinating parameters. Overall, the gene expression of two characteristic myelin proteins of the peripheral nervous system (PNS), the glycoprotein P0 and the PMP22, significantly dropped down (at least *P*<0.05) in SCs exposed to EMF at 2, 6 and 24 h (Figure 3a). In accordance, also the protein levels were significantly downregulated (*P*<0.05), whereas the effects were sharp at later times, 6 and 24 h post exposure (Figure 3b). Overall, the effects seemed to be larger for PMP22.

### Merlin levels are decreased in SCs exposed to EMFs

Changes in the tumor suppressor merlin are predictive of oncogenic tranformations in SCs. As expected, 2 h after EMF exposure the quantitative real-time PCR (qRT-PCR) analysis revealed a significant 25% decrease in merlin gene expression after treatment (compared to control; *P*<0.05); this primer effect was not evident at later time points (Figure 3c). In accordance, merlin protein levels were equally downregulated in SCs, as shown by the immunoblot in Figure 3d. Quantitative data confirmed a significant 25% downregulation (*P*<0.05) of merlin protein levels 2 h after EMF exposure, while no changes were observed at later time points (Figure 3d). Loss of merlin gene has been associated with defects of SC bipolar extension,^23^ in accordance with the enlarged SC morphologic phenotype observed (Figure 1b). However, the lower merlin levels showed a different cellular distribution (Figure 3e), because in the SCs exposed to the EMF merlin appeared more localized in the cytoplasm rather than in the nucleus (controls). Such an effect corroborated the loss of merlin suppressor function, which necessarily would occur through a nuclear localization.

### ERK and AKT signaling pathways are activated in SCs following EMF exposure

**Figure 4:**
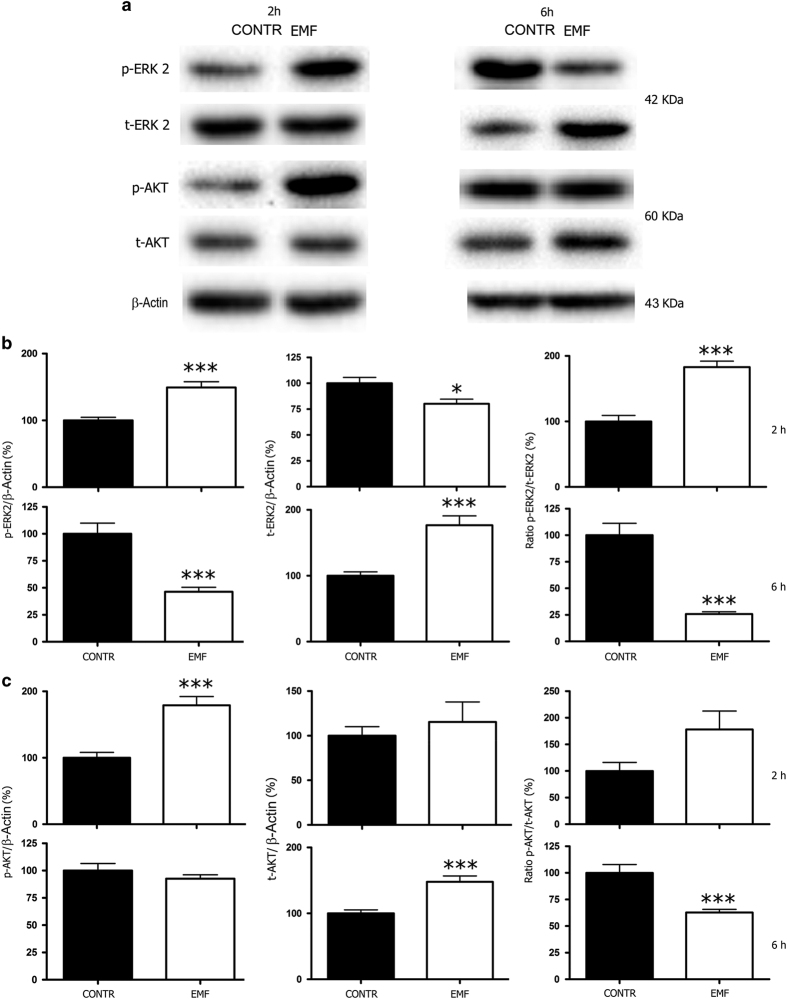
ERK and AKT signaling pathways are activated in SCs following 10 min EMF exposure. (**a**) Representative immunoblots showing the variations in phosphorylated ERK 2 (pERK 2), total ERK 2 (tERK 2), both 42 KDa, phosphorylated AKT (pAKT), total AKT (tAKT), both 60 KDa, in SCs at 2 and 6 h following EMF exposure. The *β*-actin (43 KDa) was used as a housekeeping protein. (**b**) Quantitative data at 2 h showed that pERK levels significantly increased (***P<0.001), while tERK levels decreased (*P<0.05); the pERK/tERK ratio showed that ERK signaling was activated 2 h following EMF exposure (***P<0.001, white columns). Indeed, pERK levels significantly decreased at 6 h (***P<0.001), while tERK significantly increased at 6 h (***P<0.001); pERK/tERK ratio indicated that this signaling pathway was deactivated within 6 h following EMF exposure (***P<0.001). Experiments were normalized for *β*-actin, and expressed as percentage *versus* controls (CONTR, black columns). The values are means±S.D. (*N*=3). (**c**) Quantitative data at 2 h showing that pAKT levels significantly increased (***P<0.001), while tAKT levels were unchanged; the pAKT/tAKT ratio showed an activation trend, even not significant. At 6 h, pAKT levels did not change but tAKT levels were significantly rised (***P<0.001); pAKT/tAKT ratio revealed a significant deactivation within 6 h after EMF exposure (***P<0.001, white columns). Experiments were normalized for *β*-actin, and expressed as percentage *versus* controls (CONTR, black columns). The values are means±S.D. (*N*=3). One-way ANOVA using Tukey's post-test was used for all statistical analysis.

Then we investigated the pathways involved in the SCs transformation and proliferation outcomes reported above. We found that both ERK (Ras/Raf/Mek/Erk) and AKT (PI3K/AKT) signaling pathways, which modulate biochemical pathways controlling cell growth and apoptosis, were activated. Quantitative immunoblots analysis confirmed that pERK significantly rose at 2 h (*P*<0.001), then significantly decreased at 6 h (*P*<0.001); tERK levels, indeed, showed a light but significant decrease at 2 h (*P*<0.05), then a significant increase at 6 h (*P*<0.001; Figure 4b). Analysis of pERK/tERK ratio confirmed that ERK signaling was activated at short time (2 h), and deactivated within 6 h after EMF exposure (*P*<0.001; Figure 4b).

AKT analysis showed a similar signaling profile. Quantitative immunoblots analysis confirmed pAKT significant increase at 2 h (*P*<0.001), without changes 6 h after EMF exposure; tAKT levels were significantly augmented only 6 h after EMF exposure (*P*<0.001; Figure 4c). Analysis of pAKT/tAKT ratio showed a trend of AKT activation (even not significant) at short time (2 h), and AKT significant deactivation within 6 h after EMF exposure (*P*<0.001; Figure 4c).

Taken together these data corroborate an early activation of both signaling pathways, ERK and AKT, in controlling SCs proliferation 2 h following EMF exposure. Both signaling pathways were turned off within 6 h following EMF exposure.

### Dysregulation of the Hippo signaling pathway is a consequence of EMF exposure

**Figure 5:**
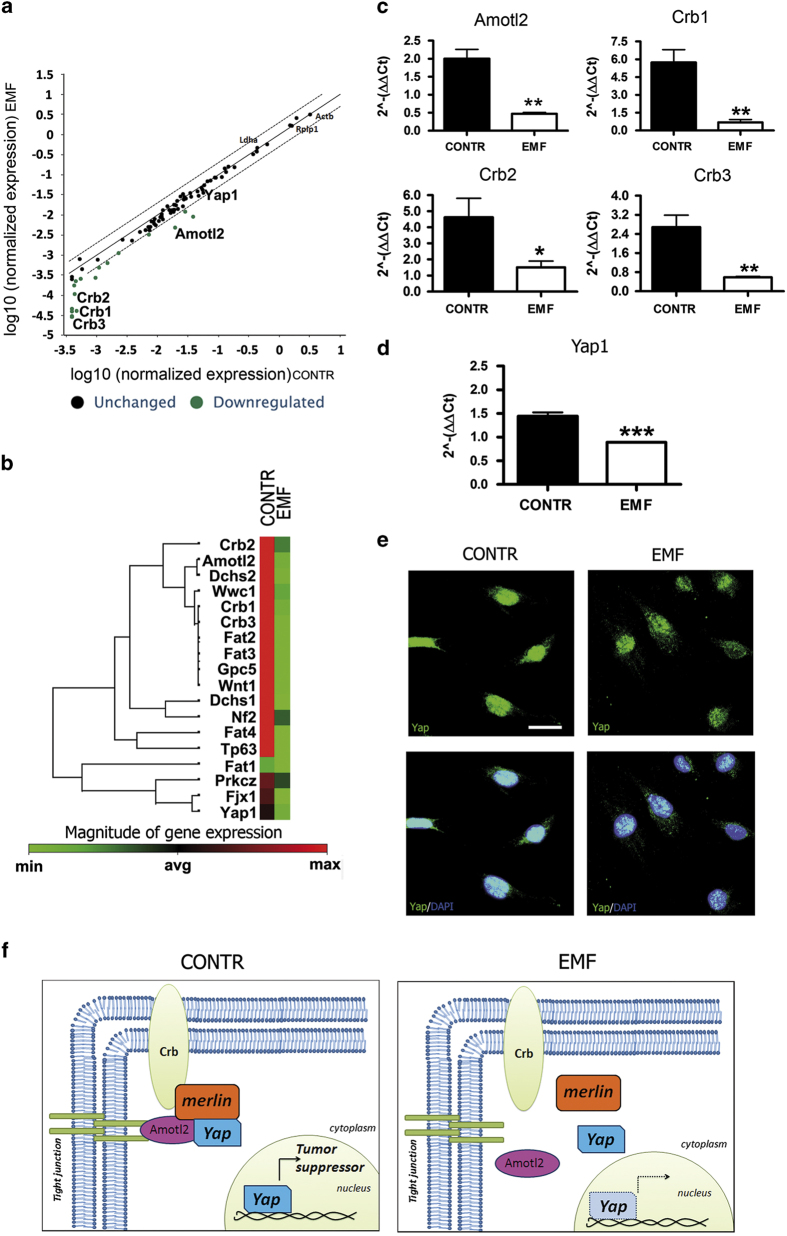
Hippo and Yap1 are altered in SCs at 2 h following EMF exposure. (**a**) Overview of the scatter plot of expression of 84 genes, related to the Hippo pathway, in which at least 21 genes were found to be changed (downregulated, green dots) in EMF-exposed SCs, *versus* controls (CONTR). The black line indicates fold changes (2^−ΔΔ^Ct) of 1.The dot lines indicate the desired fold change in gene expression threshold. Experiments were repeated at least three times, and normalized for *β*-actin (Actb), lactate dehydrogenase A (Ldha) and the 60S acidic ribosomal protein P1 (Rplp1). (**b**) Scheme of hierarchical clustering of normalized genes examined by RT^2^ profiler PCR array. Some genes coding for proteins involved in cell polarity, such as angiomotin like-2 protein (Amotl2) and Crumbs homolog proteins 1, 2 and 3 (Crb1, Crb2 and Crb3 respectively), as well as proteins involved in cell adhesion and myelinogenesis, such as dachsous proteins (Dchs), transmembrane cadherin proteins (Fat) or proto-oncogen protein1 (Wnt1), showed a decreased expression following EMF exposure (green square), *versus* controls (CONTR, red square). Also Yes-associated protein 1 (Yap1) was found to be downregulated. (**c**) Amotl2, Crb1, 2 and 3 gene expressions quantification was done by qRT-PCR, confirming a significant expression decrease following EMF exposure. Data were normalized to the housekeeping genes *β*-actin and Ldha, and expressed as difference (^ΔΔ^Ct) *versus* controls, then averaged for each experimental group. The columns control (CONTR, black) and EMF-exposed SC (EMF, white) were expressed as fold changes. The values are means±S.D. (*N*=3). One-way ANOVA using Tukey's post-test was used for statistical analysis. *P<0.05, **P<0.01. (**d**) Also Yap1 gene expressions quantification by qRT-PCR confirmed a significant decrease (***P<0.001) following EMF exposure. Data were normalized, calculated and repeated as described above. Control (CONTR, black column) and EMF-exposed SC (EMF, white column) were expressed as fold changes (means±S.D.; *N*=3). (**e**) Confocal images showed a different cellular distribution of Nf2/merlin. SCs were immunopositive for the specific markers P0 (red) and Nf2 (green). Merge images (yellow) revealed that merlin was mostly localized in the cytoplasm of EMF-exposed SCs, rather than in the nucleus (CONTR). Scale bar 10 *μ*m. (**f**) Model of the Hippo/Yap pathway involvement. In control SCs, Yap functions as tumor suppressor, assembling with Amotl2, Crb and merlin to maintain the tight junctions. In EMF-exposed SCs, the tight-junction complex is disassembled and Yap is more localized in the cytoplasm.

The RT^2^ profiler PCR array was then performed to study also the possible involvement of Hippo pathway in SCs exposed to EMF. Overall, at least 21 genes that are upstream or downstream mediators of Hippo pathway were found to be changed. Bioinformatic analysis, however, revealed a downregulation of some of these genes, differently involved in the whole Hippo pathway (Figure 5a). In detail, some proteins especially involved in cell polarity, such as Amotl2 (angiomotin like-2 protein) and Crb (Crumbs homolog) protein complex (including proteins 1, 2 and 3), showed a decrease in gene expression (Figure 5b). As expected, merlin (*Nf2*) expression was decreased, in accordance with qRT-PCR and immunoblot results previously performed (Figures 3c and d). Other proteins, such as Dchs (dachsous protein, a member of transmembrane proteins belonging to the cadherin superfamily), Fat (transmembrane cadherin proteins) or Wnt1 (proto-oncogen protein1), involved in cell adhesion and myelinogenesis respectively, were also found to be changed (Figure 5b).

Based on these results, we focused our attention on some of these genes. In particular, we were interested in Amotl2 and Crb (1, 2, 3) two proteins involved in cell morphologic rearrangements and polarity changes, as part of the tight-junction complex. Validation by qRT-PCR of the analysis previously performed in SC confirmed a significant downregulation (at least *P*<0.05) of their expressions following EMF exposure (Figure 5c). Then we analyzed the transcriptional co-activator Yap1, which is a downstream effector of Hippo pathway related to Crb/Amotl tight junction.^8^ The qRT-PCR analysis confirmed a significant downregulation (*P*<0.001) of Yap1 in SCs after EMF exposure (Figure 5d). Interestingly, Yap1 cellular localization was also changed. In control, SCs Yap1 was mainly localized in the nucleus, supporting its physiologic activity in controlling proliferation and apoptosis,^8^ while in exposed SCs its nuclear localization was decreased, being Yap1 mostly present in the cytoplasm (Figure 5e). In accordance, the absence of apoptosis in EMF-exposed SCs was strengthened by the lack of characteristic apoptotic nuclei, as observed by immunofluorescence analysis (see details in Figure 5e).

Taken together our findings, obtained by independent methods that consider different targets and mediators, supported the Hippo pathway involvement downstream to the merlin changes in SCs. This may be responsible of the Yap-mediated anti-apoptotic and proliferative effects observed on SCs (Figure 5f).

## Discussion

Our findings show that the tumor suppressor Nf2/merlin is downregulated in SCs exposed to EMFs, resulting in altered morphology, proliferation, migration and SCs myelinating capability. All of these findings are associated with a consistent modulation of ERK/AKT and Hippo signaling pathways.

These conclusions are based on some observational changes in SCs phenotype. We found that SCs change their shape from spindle-shaped to an enlarged morphology (Figure 1b), suggesting that EMFs alter the differentiation program.

It is known that during differentiation SCs switch from flat into a spindle-shaped phenotype, which presents a re-organization of f-actin cytoskeleton and the appearance of the stress fiber bundles.^24^ SCs assuming the bipolar-shaped morphology resemble the cells starting the myelination process *in vivo*.^24,25^ Therefore, our morphologic changes point to a SC de-differentiation process. Notably, this phenomenon matches with a specific increase in cell proliferation (Figure 1d). It has been hypothesized that an autocrine growth factor and/or the translocation/recycling of membrane receptors may be responsible for the SC proliferation changes. Interestingly, the transport to the plasma membrane of the *γ*-aminobutyric type B1 (GABA-B1) receptor and the neuronal excitatory amino acid transporter EAAC1/EAAT3, which localize in the elongated SC tips, appears to be associated with cytoskeleton rearrangements required for the GABAergic functions in SCs.^25,26^ However, changes in proliferation, but not in survival, were found also in other cell systems, presumably due to growth factors such as FGF-1 or IL-2^27,28^, as well as to cellular signaling factors such as ErbB4.^29^

The balance between proliferating and myelinating challenges in SCs may regulate their development toward the myelinating and/or the non-myelinating phenotype.^30,31^ In this light, an increase in proliferation/migration should correspond to a decrease in myelinating parameters. The changes in P0 and PMP22 protein expressions shown herein are in line with this hypothesis (Figures 3a and b). Moreover, the decrease in the proto-oncogen protein Wnt1 (Figure 5b) fits with the diminished P0 and PMP22 levels,^32^ corroborating the reversion in the myelinating program in the SC exposed to the EMF.

The analysis of expression and phosphorylation of ERK and AKT revealed an activation of this pathways (Figure 4), raising the possibility that EMF affects SC proliferation via mechanisms involving the ERK/AKT signaling pathways. This possibility is in agreement with previous observations obtained in schwannomas cells, in which merlin is lost while the ERK pathway is activated.^33^

Merlin is an important tumor suppressor factor, acting at the cell-to-cell tight junctions to ensure contact inhibition of growth, thus repressing proliferation.^34^ Merlin was proved to inhibit mitogenic signaling by interacting with different target effectors at the cell membrane surface.^35,36^ The EMF ability to alter membrane integrity^37^ may cause tight-junctions remodeling, inducing mitogenic signals via ERK. The reduced levels of Crb and Amotl (Figures 5b and c) suggest an impairment in tight-junction protein complex stability, entailing a dissociation of the Amotl/merlin complex (Figure 5f). If this holds true, then we suggest that merlin relocates to the cytoplasm (Figure 5e), likely promoting ERK activation and cell proliferation, as demonstrated by others.^34,38^ Altogether, these findings suggest that the tight junctions are an important target altered in EMF-exposed SCs, then leading to the loss of adhesion properties and to the increase in migrating capability (Figure 2). This is supported by the evidence that a disorganization in the cell membrane architecture abolishes the EMF effects on SCs migration (Figures 2c and d). Therefore, in line with the increased migrating capability in SCs, the decreased levels of merlin may be predictive of the oncogenic transformation.

Several lines of evidence from the literature indicate that the Hippo pathway is critical for the regulation of the tight-junction complex.^37^ Interestingly, in our study we also found an important modulation of the Hippo/Yap pathway in SCs, which contributes to the tight-junction complex and, when activated, it serves as tumor suppressor to limit cell growth. The dysregulation of the Hippo downstream effector Yap, that we found downregulated in our experiments (Figure 5d), produces an increased cell proliferation and decreased differentiation,^8^ in line with our findings. It is also important to underline that AKT promotes Yap cytosolic localization, resulting in its loss from the nucleus.^39^ Interestingly, in our experiments we found increased AKT levels associated with higher Yap immunopositivity in the cytoplasm (Figure 5e).

**Figure 6:**
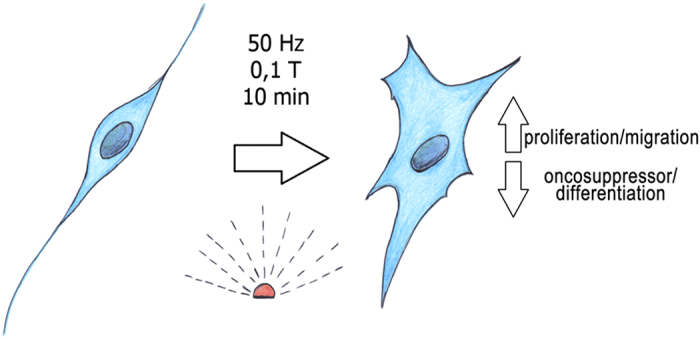
Scheme of the effects occurring in SCs following 10 min EMF exposure. The SCs that were exposed to a high-frequency (50 Hz, 0.1 T) EMF change some biological processes, mainly increasing proliferation and migration; they become more responsive to chemoattractants (see Results). Contemporarily, SCs decrease their oncosuppressor characteristic, because their levels of the oncosuppressor Nf2/merlin resulted downregulated and differently distributed (see Results). By this way, SCs alter their differentiation program, becoming, in principle, pathologically relevant for schwannoma development.

In conclusion, here we show a coordinated series of changes (i.e., tight-junction alterations, merlin decrease, SC migration, chemoresponsivity increase, Yap downregulation and redistribution), all converging into determining a reduction of the Hippo pathway involvement in the control of SCs fate following 50 Hz EMF exposure (see Figure 6). Notably, the reduction of the oncosuppressor properties in the SCs, resulting in an altered differentiation program, may be, at least in theory, pathologically relevant for the schwannoma transformation. The identification of such altered mechanisms opens new questions on the exposure of SCs to the EMFs, although the fine identification of the intracellular signaling involved deserves further investigation. It is important to note that even though the therapeutic use of low-frequency (20 Hz) EMFs has long been proposed to promote peripheral nerve regeneration,^19–21^ the 50 Hz frequency (applied in our experiments) is commonly used in several medical devices for magnetotheraphy,^40,41^ and in other electric devices. We suggest that when SCs are altered by EMF exposure, the risk to develop a neurofibroma may rise. Although the increased risk of vestibular schwannomas, coming from the long-term use of wireless phones, is still debated and needs further investigations, data from clinical case–control studies corroborate this pathogenic correlation^15–18^ and open new questions that need to be resolved.

## Materials and Methods

### Cell cultures

SC cultures were obtained by the method of Brockes^26^ with minor modifications.^27^ Sciatic nerves from 3-day-old rats were digested with 1% collagenase and 0.25% trypsin (Sigma, Milano, Italy), then cell pellets were suspended in Dulbecco's modified Eagle's medium (DMEM, Serotec, Oxford, UK), supplemented with 10% FCS (Gibco-Life Technologies, Monza, Italy) and plated onto 35 mm Petri dishes. Twenty-four hours after plating, the medium was added with 10 *μ*M arabinoside C (Sigma), then after 48 h the cultures were treated with a stream of cold DMEM-FCS 10%. Immunopanning for final purification was carried out with mouse anti-rat -Thy1.1 antibody (Serotec, Oxford, UK) followed by 500 *μ*l of baby rabbit complement (Cedarlane, Burlington, ON, Canada). Cell suspension was seeded on 60 mm Petri dishes or multiwells, maintained in DMEM, 10% FCS, 2 *μ*M forskolin, 200 *μ*g/ml bovine pituitary extract (BPE, Invitrogen, Monza, Italy). At third *in vitro* passage, SCs were treated for 48 h with 4 *μ*M forskolin, then used for different assays. SaOS-2 is a mature osteoblastic cell line derived from a human osteosarcoma. These cells were obtained from the American Type Culture Collection (Manassas, VA, USA) and routinely grown at 37 °C (5% CO_2_ and 95% air), in DMEM supplemented with 10% FCS, 2 mM glutamine, 100 IU/ml penicillin, 100 *μ*g/ml streptomycin and 1 mM sodium pyruvate.

### EMF treatment

SCs were plated and treated with EMF at 50 Hz and 0.1 Tesla (T) for 10 min at 37 °C, then were used at different time points, according to the specific experimental assays. SCs used as control were plated in same culture conditions, without EMF exposure.

### Proliferation assay

Proliferation assay on SCs was performed using an automated cell counter (Luna; Logos Biosystems Inc, Annandale, VA, USA); 10 000 cells were plated into 35 mm petri dishes and collected after 6, 24, 48 and 72 h, with Trypsin 0.05%-EDTA 0.02% in phosphate buffer solution (PBS, Euroclone, Pero, Italy). The cells suspended in DMEM were then counted with the vital stain Trypan Blue (Logos Biosystems), which stains only death cells. SaOS-2 cell proliferation was evaluated using the automated Luna cell counter (as above) and by the MTT (3-(4,5-dimethylthiazol-2-yl)-2,5-diphenyltetrazolium bromide) assay. Cells were seeded in 35 mm Petri dishes for 2 and 24 h, then stained with MTT solution (0.5 mg/ml) for 30 min at 37 °C. Absorbance was measured at 570 nm. Each experimental point was in quadruplicate and experiments replicated at least three times.

### Migration and chemotaxis assays

Wound healing assay was performed by making a scratch on the bottom of the petri, then the medium was changed with fresh medium plus mitomycin 50 ng/ml (Sigma) to counteract cell proliferation and the cells exposed to EMF. Some samples were also pre-treated with cyclodextrin 5 mM 30 min before EMF exposure. Cells were then photographed with a scanning microscope (Axiovert 200 Zeiss, Jena, Germany) at different time points: 2, 6, 24, 48 and 72 h after the scratch. Pictures were acquired using MetaVue software (Molecular Devices, Sunnyvale, CA, USA) and the distances between cell fronts were measured with Image-ProPlus 6.0 (MediaCybernetics, Bethesda, MA, USA), considering at least nine measurements from the top to the bottom. Migration was also tested by Boyden assay (chemotaxis) using a 48-well Boyden's chamber, according to the manufacturer's instructions (Neuroprobe, Cabin John, MD, USA). Cells were exposed to EMF as described above, also in the presence of cyclodextrin 5 mM. In all, 28 *μ*l of control medium (DMEM) or DMEM/FCS 1% was placed in the lower compartment of the chamber, as chemo-attractants. The open-bottom wells of the upper compartment were filled with cells (10^5^ cells/well), collected by trypsin and suspended in DMEM+0.1% bovine serum albumin (BSA, Sigma). Cells migrate through a polyvinylpyrrolidone-free polycarbonate porous membrane (8 *μ*m pores) pre-coated with gelatine (0.2 mg/ml in PBS, 5 days at 4 °C). After migration (overnight, 37 °C), cells adherent to the underside of the membrane were fixed by methanol and stained according to the Diff-Quik kit (Biomap, Milan, Italy). For quantitative analysis, cells were observed and counted using a ×40 objective on an optical microscope. Three random objective fields were counted for each well and the mean number of migrating cells was calculated.

### Immunofluorescence and confocal scanner laser microscopy

Cells were seeded on slides, then fixed in 4% paraformaldehyde (PFA), and processed for immunostaining. The SC purity (more than 98%) was tested with a specific antibody against glycoprotein P0.^27^ Phalloidin-FITC staining of f-actin (1 : 250, Sigma) was used to reveal SC cytoskeleton. Slides were incubated overnight at 4 °C in PBS, 0.25% BSA, 0.1% Triton X-100 and the specific primary antibody. The primary antibodies used in this experiments were the following: specific antibody against glycoprotein P0^27^ (1 : 200) and S100 protein (1 : 200, Sigma) to assess the SCs purity (>98%); to reveal SC cytoskeleton was used phalloidin-FITC to stain f-actin (1 : 250, Sigma) and anti-Nf2 (1: 500, Santa Cruz Biotechnology Inc, Dallas, TX, USA). The following day, the slides were washed two times and incubated 2 h at room temperature with Alexa-488 (green) or Alexa-594 (red) specific secondary antibody (1 : 800, Gibco-Life Technologies). After washing, slides were mounted using Vectashield (Vector Laboratories, Burlingame, CA, USA) and nuclei stained with 4,6-diamidino-2-phenylindole (DAPI). Controls for the specificity of antibodies included a lack of primary antibodies. Confocal microscopy was carried out using a Zeiss LSM 510 System (Gottingen, Germany) and images were processed with Image Pro-Plus 6.0.

### RNA extraction and qRT-PCR

RNA samples were extracted with using Trizol (Gibco-Life Technologies) according to the manufacturer’s protocol, then quantified with NanoDrop2000 (Thermo Scientific, Waltham, MA, USA). Pure RNA was obtained after DNAse treatment with a specific kit (Sigma). The retro-transcription reaction was carried with RT Iscript Supermix 5x (Bio-Rad, Segrate, Italy) on 1 *μ*g of purified RNA and the product was used to make qRT-PCR assay. Primers were designed by QuantPrime software (AG Bioinformatics Max-Planck, Postdam, Germany). The following primers were used: P0: 5′-CCTGCTCTTCTCTTCTTTG-3′ and 5′-CACAGCACCATAGACTTC-3′; PMP22: 5′-TCCTGTTCCTTCACATCG-3′ and 5′-TGCCAGAGATCAGTCCTG-3′; Nf2: 5′-ACGATGGCCAATGAAGCTCTGATG-3′ and 5′-TGGCCTTGATTCGCTGCATCTC-3′; *α*-tubulin: 5′-TCGCGCTGTAAGAAGCAACACC-3′ and 5′-GGAGATACACTCACGCATGGTTGC-3′; *β*2-microglobulin: 5′-TGCTTGCAGAGTTAAACACGTCAC-3′ and 5′-TTACATGTCTCGGTCCCAGGTG-3′; *α*-tubulin and *β*2-microglobulin were used as housekeepers. qRT-PCR was performed by measuring the incorporation of SYBR Green dye (Bio-Rad) on CFX 96 Real Time System-C1000 touch thermal cycler (Bio-Rad). Data analysis was performed using the CFX Manager 2.0 software (Bio-Rad), based on ^ΔΔ^Ct method for the relative quantification. The threshold cycle number (Ct) values of both the calibrator and the samples of interest were normalized to the Ct of the endogenous housekeeping genes. As calibrator we used the RNA obtained from control samples.

### Expression profile of Hippo pathway

The Rat Hippo signaling pathway RT2 Profiler PCR arrays (SuperArray Bioscience, Qiagen, Valencia, CA, USA) were used to profile the expression of 84 genes related to Hippo signaling pathway. Total RNA was extracted from SCs, 2 h after treatment, as above, and their controls with the same method. Single-stranded cDNA was synthesized from 2 *μ*g of total RNA by using the SuperArray reaction ready first-strand cDNA synthesis kit. The cDNAs were mixed with SuperArray RT2 Real time SYBR Green/ROX PCR master mix and real-time PCR performed in accordance with the manufacturer’s instructions. Thermal cycling and fluorescence detection were performed using an ABI Prism 7700 Sequence Detection System (Applied Biosystems, Monza, Italy), then the expression of Hippo signaling regulated transcripts was compared between groups.

### Western blot

Protein samples were extracted in lysis buffer (PBS, 1% Nonidet P-40 and 1 mM EDTA; all from Sigma) containing a cocktail of protease inhibitors (Sigma). Samples were heated 20 min at 55 °C to denaturate secondary structures, then 15 *μ*g was loaded onto a SDS-PAGE gel (Criterion TGX, Bio-Rad) and run at 200 V for 40 min in running buffer. Gels were electroblotted to Hybond nitrocellulose membrane (GE Healthcare, Milano, Italy). Membranes were blocked with 5% not-fat dry milk (Bio-Rad) in PBS (Euroclone) before incubation with the primary antibody diluted in the blocking solution. Primary antibodies used were rabbit anti-P0 (1 : 300, Sigma-Genosys, Spring, TX, USA), rabbit anti-PMP22 (1 : 300, Abcam, Cambridge, UK), anti-Nf2 (1 : 100, Santa Cruz Biotech, Dallas, TX, USA) and monoclonal anti-alpha-tubulin as reference (1 : 500, Sigma). To detect ERK2 and AKT levels, 10 *μ*g proteins were loaded and the antibodies used were the following: anti-phospho-AKT Ser473 (1 : 1000, Cell Signaling Technology, Danvers, MA, USA), anti-AKT (1 : 1000, Cell Signaling Technology), anti-phospho-ERK2 Tyr185/187 (1 : 1000, Cell Signaling Technology), anti-ERK2 (1 : 5000 Santa Cruz Biotechnology), anti-*β*-Actin (1 : 10 000, Sigma-Aldrich, Milano, Italy). In this case, results were standardized using *β*-actin as a reference. Membranes were incubated with appropriated HRP-conjugated secondary antibodies (Millipore, Temecula, CA, USA). Immunocomplexes were revealed by enhanced chemiluminescence (ECL; GE Healthcare), visualized using the Chemidoc MP Imaging System (Bio-Rad) and analyzed by the Image Lab software (Bio-Rad).

### Statistical analysis

Data were statistically evaluated by GraphPad Prism 4.00 (San Diego, CA, USA). Statistical significance between groups was determined by means of an unpaired Student’s *t*-test, one-way ANOVA with Tukey's post-test, or by two-way ANOVA using Bonferroni’s *post hoc* test. *P*-values <0.05 were considered as significant.

## Abbreviations

*Nf2*: neurofibromin type 2 gene
SC: Schwann cell
EMF: electromagnetic field
ERM: ezrin-radixin-moesin
Yap: Yes-associated protein
P0: myelin protein zero
FCS: fetal calf serum
PNS: peripheral nervous system
pERK: phosphorylated ERK
tERK: total ERK
pAKT: phosphorylated AKT
tAKT: total AKT
Amotl2: angiomotin like-2 protein
Crb: Crumbs homolog
Dchs: dachsous protein
Fat: transmembrane cadherin proteins
Wnt1: proto-oncogen protein1
DMEM: Dulbecco's modified Eagle's medium
MTT: 3-(4,5-dimethylthiazol-2-yl)-2,5-diphenyltetrazolium bromide
BPE: bovine pituitary extract
BSA: bovine serum albumin
PBS: phosphate buffer solution
PFA: paraformaldehyde
DAPI: 4,6-diamidino-2-phenylindole.
