# Supplementary figures and images for: Tumor suppressor Nf2/merlin drives Schwann cell changes following electromagnetic field exposure through Hippo-dependent mechanisms
Source: Cell Death Discov. 2015 Sep 7;1:15021–. doi: 10.1038/cddiscovery.2015.21 (PMC4979489; doi:10.1038/cddiscovery.2015.21)

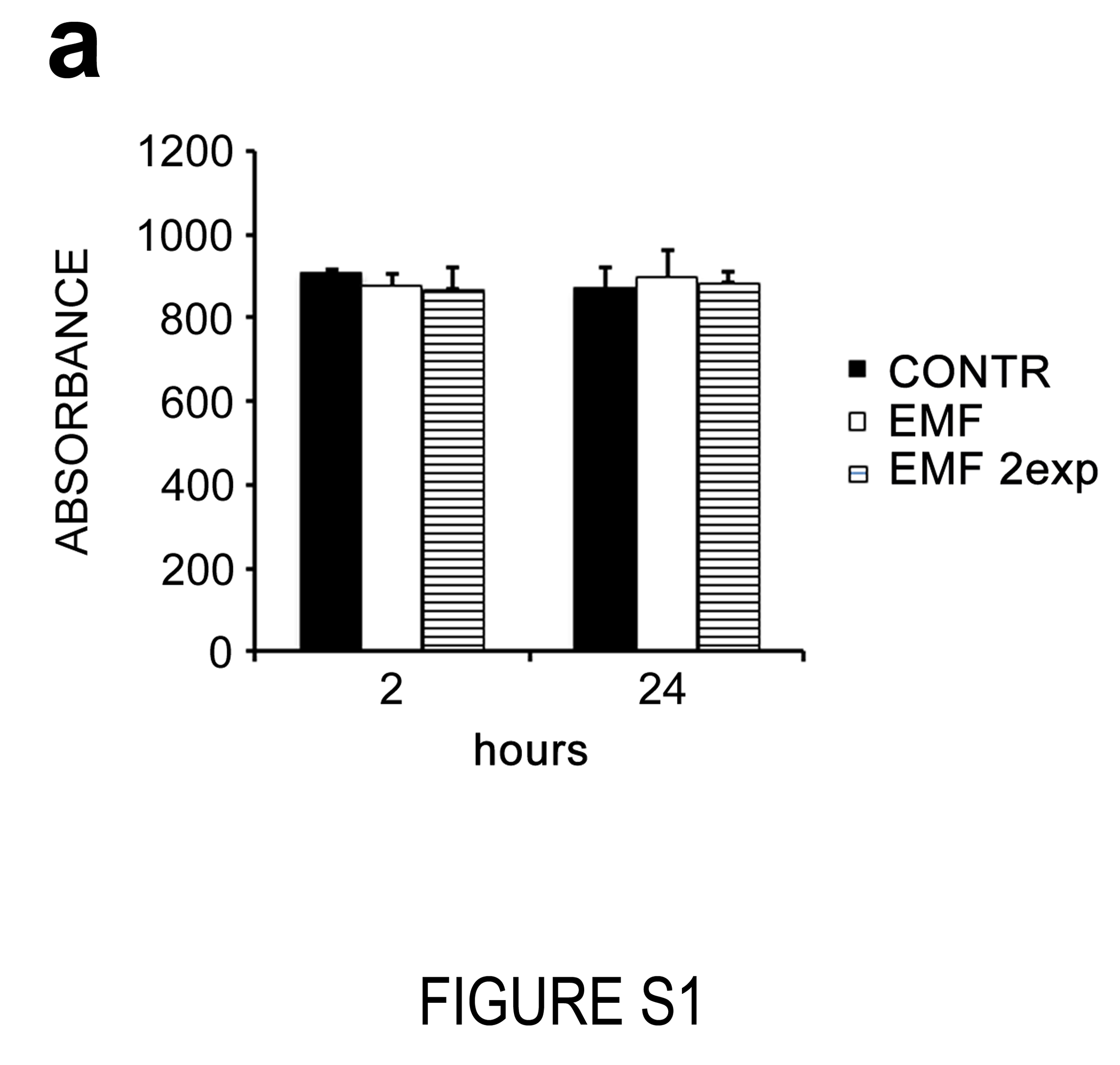

Supplement: Supplementary Figure S1 [file cddiscovery201521-s1.jpg]
