# Supplementary material for: Tumor suppressor Nf2/merlin drives Schwann cell changes following electromagnetic field exposure through Hippo-dependent mechanisms
Source: Cell Death Discov. 2015 Sep 7;1:15021–. doi: 10.1038/cddiscovery.2015.21 (PMC4979489; doi:10.1038/cddiscovery.2015.21)
Supplement: Supplementary Figure S1 Legend [file cddiscovery201521-s2.doc]

**Supplementary Figure Legends**

**Supplementary Figure S1. Assessment of SaOS-2 cell migration following EMF exposure.** (a) SaOS-2 cell proliferation was assessed at 2 and 24 hours following a single (white columns) or double (lined columns) EMF exposure. EMFs did not produce any significant change in cell proliferation. Experiments were repeated at least three times and data are expressed as absorbance at 570 nM following MTT assay
